# Carbapenems versus non-carbapenems as definitive treatment for hypervirulent *Klebsiella pneumoniae* bacteremia in Taiwan

**DOI:** 10.1128/aac.00846-25

**Published:** 2025-11-18

**Authors:** Chih-Han Juan, Sheng-Hua Chou, Chien Chuang, Shih-Neng Lin, Xin-Ni Wu, Szu-Yu Liu, Yu-Chien Ho, Yi-Tsung Lin

**Affiliations:** 1 Division of Infectious Diseases, Department of Medicine, Taipei Veterans General Hospital 46615, Taipei, Taiwan; 2 Institute of Emergency and Critical Care Medicine, National Yang Ming Chiao Tung University 390197 , Taipei, Taiwan; 3 School of Medicine, National Yang Ming Chiao Tung University 390197 , Taipei, Taiwan; 4 Department of Rehabilitation, Taoyuan General Hospital, Ministry of Health and Welfare 63487, Taoyuan, Taiwan; 5 Center for Infection Control, Taipei Veterans General Hospital 46615, Taipei, Taiwan; University of California, San Francisco, California, USA

**Keywords:** hypervirulent, *Klebsiella pneumoniae*, bacteremia, carbapenem

## Abstract

Hypervirulent *Klebsiella pneumoniae* (hvKp) can cause serious community-acquired pyogenic infections and is prevalent in Asian countries. Unlike classical *K. pneumoniae*, which is usually multidrug resistant, hvKp is usually antimicrobial-sensitive. However, the optimal definitive treatment for hvKp bacteremia remains unclear, with carbapenems often used due to severe conditions. We aimed to compare clinical outcomes of patients with hvKp bacteremia treated with carbapenems and non-carbapenems in Taiwan. This retrospective study included patients with monomicrobial hvKp bacteremia treated with appropriate definitive therapy at Taipei Veterans General Hospital from January 2015 to December 2017. *K. pneumoniae* isolates carrying *rmpA*/*rmpA2* genes were defined as hypervirulent strains. A multivariate logistic regression model and propensity score-matched analyses were used to identify independent risk factors for 28-day mortality. A total of 236 patients with hvKp bacteremia were identified, and 52 patients received carbapenems. The non-carbapenem group had a lower 28-day mortality than that in the carbapenem group (10.3% versus 23.1%, *P* = 0.016). Multivariate analysis showed definitive carbapenem therapy was not associated with 28-day mortality (OR, 1.39; 95% CI, 0.47–4.10; *P* = 0.549). Among 90 patients with an Acute Physiology and Chronic Health Evaluation (APACHE) II score ≥15, definitive therapy with carbapenems was still not associated with 28-day mortality (OR, 2.63; 95% CI, 0.58 to 12.00; *P* = 0.212). The propensity score-matched analyses yielded similar results. In conclusion, definitive carbapenem therapy for hvKp bacteremia was not associated with a survival benefit compared with non-carbapenem therapy, even in critically ill patients. The study provides valuable insights into antimicrobial stewardship in endemic regions of hvKp.

## INTRODUCTION

*Klebsiella pneumoniae* is a human commensal pathogen, but it can cause a variety of infections, such as pneumonia, urinary tract infection, intra-abdominal infections, bacteremia, etc. (1). It is classified into two pathotypes: hypervirulent *K. pneumoniae* (hvKp) and classical *K. pneumoniae* (cKp) (2).

The cKp strains are widely investigated worldwide and known to cause infections in hosts with comorbidities or immunocompromised conditions in healthcare settings. The cKp strains can easily acquire antibiotic resistance genes and cause critical hospital outbreaks (2). By contrast, the hvKp strains have been prevalent in East Asian countries and have emerged in Western countries recently (2, 3). Unlike the cKP strains, the hvKP strains are typically susceptible to most antimicrobials, with the exception of ampicillin. It can lead to life-threatening metastatic diseases, such as bacteremia, liver abscess, pneumonia, endophthalmitis, meningitis, and necrotizing fasciitis, in immunocompetent individuals from the community (2). In the past, virulence and carbapenem resistance evolved independently in two distinct clonal lineages of *K. pneumoniae*. However, the convergence of these evolutionary pathways has led to the emergence of strains that are both carbapenem-resistant and hypervirulent (2, 3).

Management of infections caused by hvKp strains requires the prompt initiation of antibiotic therapy and appropriate source control (2, 4). Research on the definitive antibiotic treatment of hvKp infections usually originates from patients with liver abscesses in endemic regions of hvKp (5, 6). In Taiwan, our group previously reported that fluoroquinolones demonstrated comparable clinical efficacy to β-lactams as the major therapy for treatment of *K. pneumoniae* liver abscess, with 94% of strains classified as hvKp strains (7). Except for bacteremic liver abscess, reports on the definitive antibiotics for hvKp bacteremia are limited (8–10). Despite hvKp strains being usually antimicrobial-susceptible, carbapenem is commonly used as definitive treatment for hvKp bacteremia because of high disease severity (8–10). One previous study in Japan demonstrated that the proportion of carbapenems as definitive therapy against hvKp bacteremia was significantly higher than that of other antimicrobial agents (8). In Western countries, research on treatment experiences for infections caused by hvKp strains is limited, and carbapenems are also commonly used (10, 11).

To date, the clinical efficacy of different definitive antibiotics for hvKp bacteremia is uncertain. The widely used carbapenems would pose a notable challenge to antimicrobial stewardship. This study aimed to compare the clinical outcomes of patients with hvKp bacteremia, treated with carbapenems and non-carbapenems as definitive antibiotics.

## MATERIALS AND METHODS

### Study design and data collection

This retrospective study included consecutive patients with hvKp bacteremia at Taipei Veterans General Hospital, a 2,900-bed tertiary-care teaching hospital in Taiwan, from January 2015 to December 2017. For patients with multiple *K. pneumoniae* bacteremia episodes, only the first episode was included. Patients were excluded if they were under the age of 20, had polymicrobial bacteremia, died prior to definitive treatment, or received inappropriate definitive treatment. The following information was obtained from the electronic medical records of eligible patients: demographic characteristics of the patients, location at the time of culture, source of bacteremia, co-morbidities, immunosuppression, surgeries, invasive procedures or devices, surgical drainage, mechanical ventilation, antimicrobial therapy, severity of illness, outcome, and mortality. The study protocol was approved by the institutional review board of Taipei Veterans General Hospital.

### Definitions and outcomes

Community-acquired bacteremia is defined as hvKp isolates identified in patients upon admission or within 48 hours of admission who did not fit the criteria for healthcare-associated bacteremia (12). Healthcare-associated bacteremia is defined as hvKp isolates identified in patients upon admission or within 48 hours of admission meeting any of the following criteria (12): having received intravenous therapy at home or in an outpatient clinic within the last 30 days; having received renal dialysis in a hospital or clinic within the last 30 days; having been hospitalized for 2 or more days within the last 90 days; or having resided in a nursing home or long-term care facility. Hospital-acquired bacteremia is defined as hvKp isolates identified in patients more than 48 hours after admission (12). Prior antibiotic exposure is defined as at least 2 days of therapy within 30 days prior to acquiring bacteremia. Requirement of intensive care unit (ICU) admission was defined as admission to the ICU within 7 days of the onset of bacteremia. We used the Pitt bacteremia score and the Acute Physiology and Chronic Health Evaluation (APACHE) II score to determine the severity of illness within 24 hours of the onset of bacteremia (13).

Appropriate empirical antimicrobial therapy is defined as the administration of at least one antimicrobial agent to which the causative pathogen is susceptible within 24 hours of the onset of bacteremia at the approved route and dosage for at least 2 days. Appropriate definite antimicrobial therapy is defined as the administration of at least one antimicrobial agent to which the causative pathogen is susceptible within 24 hours of the available antimicrobial susceptibility test at the approved route and dosage. The group of appropriate definitive treatment (carbapenems or not) was defined as antibiotics used for the majority of the overall therapy duration after the antimicrobial susceptibility result is available. The major interest of this study was 28-day all-cause mortality from the time of the index positive blood culture.

### Microbiological methods

All *K. pneumoniae* isolates were identified using matrix-assisted laser desorption-ionization time-of-flight mass spectrometry (bioMérieux, Marcy-l’Etoile, France) or the VITEK2 system (bioMérieux, Marcy-l’Étoile, France). Antimicrobial susceptibility to *K. pneumoniae* isolates was determined using the VITEK2 system (bioMérieux, Marcy-l’Etoile, France) and interpreted according to the guidelines of the Clinical and Laboratory Standards Institute (14). Multidrug-resistant (MDR) *K. pneumoniae* isolate is defined as non-susceptibility to at least one agent in three or more antimicrobial categories (15). We conducted cps genotyping of *K. pneumoniae* isolates to identify capsular genotypes, utilizing polymerase chain reaction targeting K-serotype-specific alleles at the wzy loci, including serotypes K1, K2, K5, K20, K54, and K57 (16). The detection of *rmpA* and *rmpA2* genes was performed as described previously (16). HvKp strains were defined as those with *rmpA* or *rmpA2* genes (17).

### Statistical analyses

Categorical variables were compared using the chi-square or Fisher’s exact test. The analyses of continuous variables were conducted using the Student’s *t* test and Mann–Whitney U test (Wilcoxon rank-sum test). To adjust for treatment selection bias, propensity scores were calculated, including age, gender, location of infection acquisition, source of infection, disseminated infections, underlying diseases, Charlson’s comorbidity index, invasive procedures and devices at onset of bacteremia, surgery within 2 weeks, prior antibiotic exposure, source control, requirement of ICU admission, Pitt bacteremia score, APACHE II score, appropriate empirical antimicrobial therapy, septic shock when bacteremia, and respiratory failure requiring mechanical ventilation.

To identify independent predictors for 28-day all-cause mortality of hvKp bacteremia, we used multivariate logistic regression analysis. All biologically possible variables with *P* values of <0.05 in univariate analyses were incorporated into a model using an enter approach. Further analyses were conducted using propensity score matching with the nearest-neighbor method and replacement, in which patients who received carbapenems were matched in a 1:2 ratio to those who received non-carbapenems.

A two-tailed *P* value <0.05 was considered statistically significant. All statistical analyses were performed using the Statistical Package for the Social Sciences software, version 23.0 (IBM Corp, Armonk, NY, USA) and R (R Foundation for Statistical Computing, Vienna, Austria), version 4.5.1.

## RESULTS

### Baseline characteristics of patients

**Fig 1:**
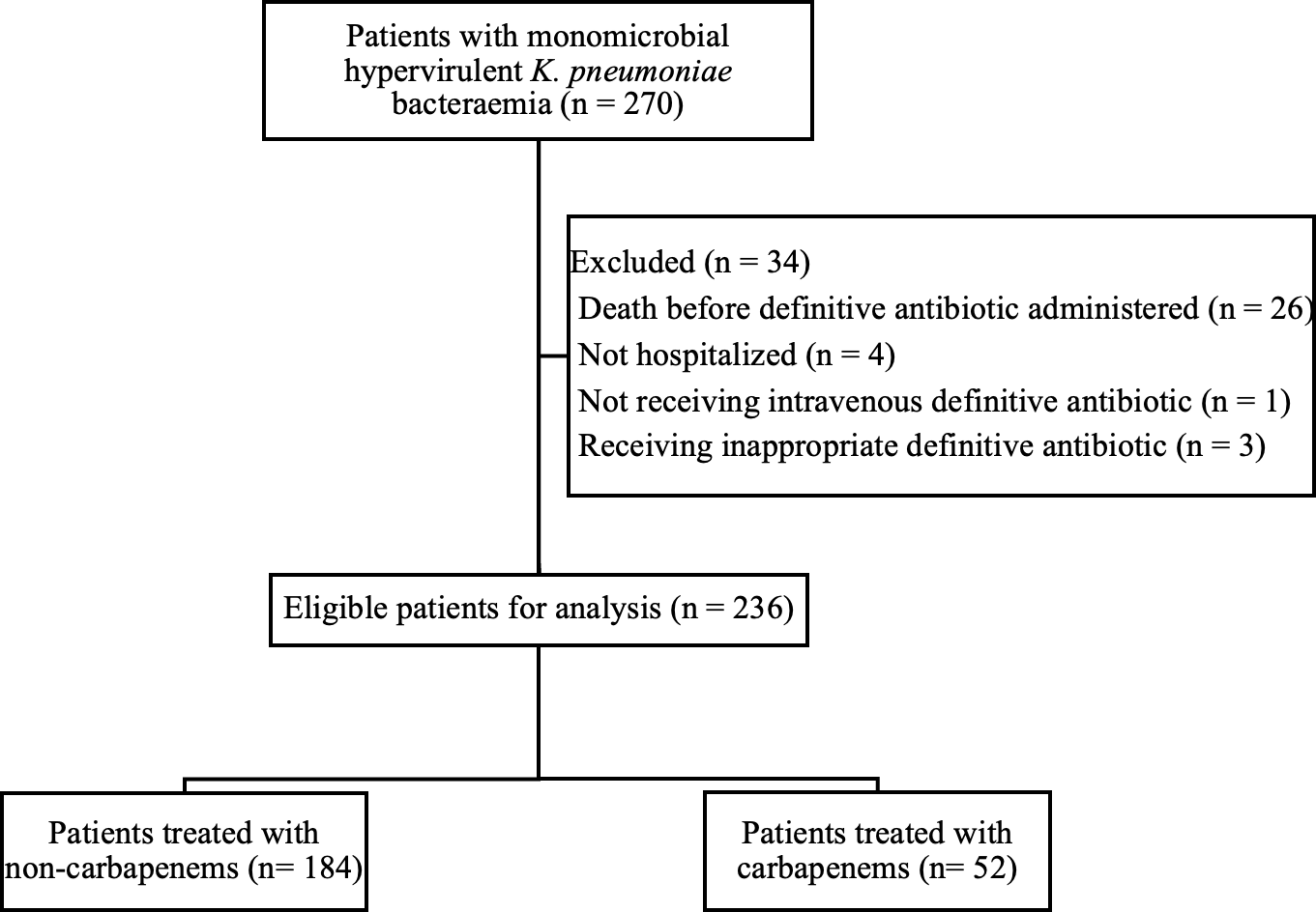
Study flowchart.

A total of 270 patients with monomicrobial hvKp bacteremia were identified, and 236 patients were eligible for analysis (Fig. 1). The median (interquartile range, IQR) age of these patients was 72.0 (60.0–81.8) years, and men were predominant (*n* = 157, 66.5%). Among them, 91 (38.6%), 72 (30.5%), and 73 (30.9%) patients had community-acquired, healthcare-associated, and hospital-acquired bacteremia, respectively. Liver abscess (*n* = 52, 22.0%), pneumonia (*n* = 52, 22.0%), and urinary tract infection (*n* = 49, 20.8%) were the common sources of bacteremia. Diabetes (*n* = 106, 44.9%), chronic kidney disease (*n* = 98, 41.5%), and malignancy (*n* = 84, 35.6%) were common comorbidities. There were 184 patients (78.0%) in the non-carbapenem group, including treatment with cephalosporin in 145, penicillin-derived antibiotic in 18, and fluoroquinolone in 21 patients. The remaining 52 patients (22.0%) were in the carbapenem group, including ertapenem in 20, meropenem in 12, imipenem-cilastatin in 15, and doripenem in 5 patients. The 14-day and 28-day mortality rates were 8.9% (*n* = 21) and 13.1% (*n* = 31), respectively.

### Comparison of clinical characteristics and outcomes of patients receiving non-carbapenems versus carbapenems

Table 1 shows the comparison of clinical characteristics between patients treated with non-carbapenems and carbapenems for hvKp bacteremia. The proportion of community-acquired bacteremia was higher in the non-carbapenem group than in the carbapenem group (42.9% versus 23.1%, *P* = 0.011), while a higher proportion of hospital-acquired bacteremia was observed in the carbapenem group than in the non-carbapenem group (48.1% versus 26.1%, *P* = 0.002). With regard to infection source, the proportion of pneumonia (32.7% versus 19.0%, *P* = 0.036) was significantly higher in the carbapenem group than in the non-carbapenem group.

**TABLE 1:** Comparison of clinical characteristics in patients with hypervirulent *K. pneumoniae* bacteremia receiving non-carbapenems versus carbapenems as definitive antibiotic^*a*^^,*b*^

	Original cohort			Propensity score-matched cohort		
	Non-carbapenems (*n* = 184)	Carbapenems (*n* = 52)	*P* value	Non-carbapenems (*n* = 104)	Carbapenems (*n* = 52)	*P* value
Demographics						
Age, median (IQR), years	69.5 (58.5–82.0)	75 (64.5–80.0)	0.144	73 (61.0–83.3)	75 (64.8–80.0)	0.620
Gender, male	124 (67.4)	33 (63.5)	0.596	68 (65.4)	33 (63.5)	>0.953
Location of infection acquisition						
Community	79 (42.9)	12 (23.1)	0.011	33 (31.7)	12 (23.1)	0.349
Healthcare associated	57 (31.0)	15 (28.8)	0.768	34 (32.7)	15 (28.8)	0.760
Hospital	48 (26.1)	25 (48.1)	0.002	37 (35.6)	25 (48.1)	0.183
Source of infection						
Respiratory system	35 (19.0)	17 (32.7)	0.036	24 (23.1)	17 (32.7)	0.274
Urinary	41 (22.3)	8 (15.4)	0.279	18 (17.3)	8 (15.4)	0.939
Intra-abdominal^*c*^	35 (19.0)	10 (19.2)	0.973	19 (18.3)	10 (19.2)	>0.999
Liver abscess	44 (23.9)	8 (15.4)	0.190	22 (21.2)	8 (15.4)	0.518
Primary bacteremia	25 (13.6)	9 (17.3)	0.500	18 (17.3)	9 (17.3)	>0.999
Skin and soft tissue	8 (4.3)	1 (1.9)	0.688	5 (4.8)	1 (1.9)	0.659
Intravenous catheter	0 (0.0)	0 (0.0)	N/A	0 (0.0)	0 (0.0)	N/A
Others	5 (2.7)	1 (1.9)	>0.999	3 (2.9)	1 (1.9)	>0.999
Disseminated infection	7 (3.8)	2 (3.8)	>0.999	5 (4.8)	2 (3.8)	>0.999
Underlying diseases						
Malignancy	65 (35.3)	19 (36.5)	0.872	42 (40.4)	19 (36.5)	0.772
Diabetes mellitus	79 (42.9)	27 (51.9)	0.250	49 (47.1)	27 (51.9)	0.692
Chronic kidney disease	66 (35.9)	32 (61.5)	0.001	46 (44.2)	32 (61.5)	0.062
Hemodialysis	9 (4.9)	9 (17.3)	0.003	7 (6.7)	9 (17.3)	0.076
Congestive heart failure	11 (6.0)	9 (17.3)	0.010	10 (9.6)	9 (17.3)	0.261
Liver cirrhosis	28 (15.2)	4 (7.7)	0.250	12 (11.5)	4 (7.7)	0.641
Cerebral vascular disease	18 (9.8)	7 (13.5)	0.447	8 (7.7)	7 (13.5)	0.387
Chronic obstructive lung disease	9 (4.9)	2 (3.8)	>0.999	7 (6.7)	2 (3.8)	0.716
Collagen vascular disease	8 (4.3)	0 (0.0)	0.205	0 (0.0)	0 (0.0)	N/A
Transplantation	0 (0.0)	1 (1.9)	0.220	0 (0.0)	1 (1.9)	0.723
Immunosuppression^*d*^	27 (14.7)	12 (23.1)	0.150	21 (20.2)	12 (23.1)	0.835
Charlson’s comorbidity index, median (IQR)	6.0 (4.0–8.0)	6.5 (5.0–9.0)	0.067	6.5 (5.0–9.0)	6.5 (5.0–9.0)	0.582
Invasive procedures and devices at onset of bacteremia						
Central venous catheter	17 (9.2)	16 (30.8)	<0.001	14 (13.5)	16 (30.8)	0.018
Nasogastric/nasojejunal tube	35 (19.0)	17 (32.7)	0.036	20 (19.2)	17 (32.7)	0.096
Urinary catheter	42 (22.8)	20 (38.5)	0.024	30 (28.8)	20 (38.5)	0.302
Endotracheal tube^*e*^	15 (8.2)	8 (15.4)	0.120	7 (6.7)	8 (15.4)	0.150
Tracheostomy	5 (2.7)	5 (9.6)	0.029	4 (3.8)	5 (9.6)	0.275
Surgical drainage	15 (8.2)	6 (11.5)	0.449	11 (10.6)	6 (11.5)	>0.999
Surgery within 2 weeks	21 (11.4)	12 (23.1)	0.032	16 (15.4)	12 (23.1)	0.338
Prior antibiotic exposure						
Any antibiotic	42 (22.8)	28 (53.8)	<0.001	29 (27.9)	28 (53.8)	0.003
First or second generation cephalosporin^*f*^	27 (14.7)	13 (25.0)	0.080	17 (16.3)	13 (25.0)	0.281
Third or fourth generation cephalosporin^*g*^	9 (4.9)	4 (7.7)	0.491	3 (2.9)	4 (7.7)	0.338
β-lactam and β-lactamase inhibitor^*h*^	14 (7.6)	11 (21.2)	0.005	11 (10.6)	11 (21.2)	0.122
Carbapenem^*i*^	9 (4.9)	6 (11.5)	0.083	6 (5.8)	6 (11.5)	0.339
Fluoroquinolone^*j*^	4 (2.2)	7 (13.5)	0.003	3 (2.9)	7 (13.5)	0.028
Aminoglycoside^*k*^	1 (0.5)	2 (3.8)	0.123	1 (1.0)	2 (3.8)	0.536
Tigecycline	1 (0.5)	5 (9.6)	0.002	1 (1.0)	5 (9.6)	0.027
Glycopeptide^*l*^	6 (3.3)	4 (7.7)	0.233	3 (2.9)	4 (7.7)	0.338
Metronidazole	7 (3.8)	3 (5.8)	0.463	5 (4.8)	3 (5.8)	>0.999
Source control	49 (26.6)	15 (28.8)	0.751	36 (34.6)	15 (28.8)	0.587
Septic shock when bacteremia	50 (27.2)	24 (46.2)	0.009	33 (31.7)	24 (46.2)	0.112
Respiratory failure requiring mechanical ventilation	21 (11.4)	7 (13.5)	0.687	12 (11.5)	7 (13.5)	0.931
Requirement of ICU admission	39 (21.2)	11 (21.2)	0.995	22 (21.2)	11 (21.2)	>0.999
Pitt bacteremia score, median (IQR)	1.0 (0.0–3.0)	2.5 (1.0–4.0)	0.006	1.0 (0.0–3.0)	2.5 (1.0–4.0)	0.024
APACHE II score, median (IQR)	11.0 (8.0–17.0)	15.0 (8.3–23.8)	<0.001	13.0 (8.8–18.0)	15.0 (8.8–23.3)	0.037

^*a*^ Data are presented as number (%) of patients, unless stated otherwise.

^*b*^ IQR, interquartile range; ICU, intensive care unit; APACHE, Acute Physiology and Chronic Health Evaluation; N/A, not applicable.

^*c*^ Intra-abdominal infection was defined as infections of single organs of the abdomen with or without extension into the peritoneal space, with exclusion of liver abscess.

^*d*^ Immunosuppression was defined as meeting one of the following criteria: neutropenia, use of corticosteroids, or receiving chemotherapy.

^*e*^ Endotracheal tube was defined as the patient being intubated at the onset of bacteremia.

^*f*^ Including cefazolin and cefuroxime.

^*g*^ Including cefoperazone, ceftriaxone, cefotaxime, cefepime, and cefpirome.

^*h*^ Including amoxicillin/clavulanate, ampicillin/sulbactam, piperacillin/tazobactam, and ticarcillin/clavulanate.

^*i*^ Including ertapenem, imipenem, meropenem, and doripenem.

^*j*^ Including ciprofloxacin, levofloxacin, and moxifloxacin.

^*k*^ Including amikacin, gentamicin, and isepamicin.

^*l*^ Including vancomycin and teicoplanin.

With regard to co-morbidities, the proportion of chronic kidney disease (61.5% versus 35.9%, *P* = 0.001), hemodialysis (17.3% versus 4.9%, *P* = 0.003), and congestive heart failure (17.3% versus 6.0%, *P* = 0.010) was observed more frequently in the carbapenem group than in the non-carbapenem group. Prior antibiotic exposure was less common in the non-carbapenem group than in the carbapenem group (22.8% versus 53.8%, *P* < 0.001). The proportion of septic shock was higher in the carbapenem group than in the non-carbapenem group (46.2% versus 27.2%, *P* = 0.009). Patients in the carbapenem group had a higher disease severity than those in the non-carbapenem group, as indicated by the median Pitt bacteremia score (2.5 versus 1.0, *P* = 0.006) and median APACHE II score (15.0 versus 11.0, *P* < 0.001).

The proportion of appropriate empirical antimicrobial therapy was higher in the non-carbapenem group than that in the carbapenem group (96.2% versus 82.7%, *P* = 0.001) (Table 2). The 28-day mortality in the non-carbapenem group was lower than that of the carbapenem group (10.3% versus 23.1%, *P* = 0.016).

**TABLE 2:** Comparison of clinical outcomes in patients with hypervirulent *K. pneumoniae* bacteremia receiving non-carbapenems versus carbapenems as definitive antibiotic^*a*^^,*b*^

	Original cohort			Propensity score-matched cohort		
	Non-carbapenems (*n* = 184)	Carbapenems (*n* = 52)	*P* value	Non-carbapenems (*n* = 104)	Carbapenems (*n* = 52)	*P* value
Appropriate empirical antimicrobial therapy	177 (96.2)	43 (82.7)	0.001	97 (93.3)	43 (82.7)	0.076
Length of hospital stay, median (IQR), days	17.5 (12.0–33.5)	28.5 (16.0–44.8)	0.007	20.0 (14.0–38.0)	28.5 (16.0–44.8)	0.092
14-day mortality	14 (7.6)	7 (13.5)	0.191	10 (9.6)	7 (13.5)	0.650
28-day mortality	19 (10.3)	12 (23.1)	0.016	14 (13.5)	12 (23.1)	0.197
In-hospital mortality	25 (13.6)	18 (34.6)	0.001	18 (17.3)	18 (34.6)	0.027

^*a*^ Data are presented as number (%) of patients, unless stated otherwise.

^*b*^ IQR, interquartile range.

### Microbiological characteristics of hvKp strains

Table S1 depicts the antimicrobial susceptibility of hvKp isolates among the two groups. HvKp isolates from the non-carbapenem group had a higher proportion of wild-type antibiotic susceptibility (isolates that are susceptible to several classes of antibiotics but not ampicillin) than those from the carbapenem group (87.5% versus 65.4%, *P* < 0.001). The proportion of MDR-hvKp isolates was higher in the carbapenem group than that from the non-carbapenem group (32.7% versus 6.0%, *P* < 0.001).

The distribution of capsular types and the presence of *rmpA*/*rmpA2* genes among all hvKp isolates are shown in Table S2. Interestingly, the six virulent capsular types (K1, K2, K5, K20, K54, and K57) were more common in the non-carbapenem group than in the carbapenem group (80.4% versus 67.3%, *P* = 0.045).

### Risk factors for 28-day mortality in patients with hvKp bacteremia

The comparison of characteristics in patients with hvKp bacteremia by survival status within 28 days is shown in Table S3. In the multivariate analysis, chronic obstructive lung disease (OR, 6.27; 95% CI, 1.12 to 34.97; *P* = 0.036) and the APACHE II score (OR, 1.07; 95% CI, 1.01 to 1.13; *P* = 0.033) were the independent risk factors for 28-day mortality. Though definitive therapy with carbapenems for hvKp bacteremia was associated with 28-day mortality in the univariate analysis, it was statistically nonsignificant in the multivariate analysis (OR, 1.39; 95% CI, 0.47 to 4.10; *P* = 0.549) (Table 3).

**TABLE 3:** Multivariate logistic regression analysis of predictors for 28-day mortality in patients with hypervirulent *K. pneumoniae* bacteremia^*a*^

Variables	Univariate analysis OR (95% CI)	*P* value	Multivariate analysis OR (95% CI)	*P* value
Definitive antibiotic				
Non-carbapenems	1.00 (reference)		1.00 (reference)	
Carbapenems	2.61 (1.17–5.80)	0.019	1.39 (0.47–4.10)	0.549
Male	0.42 (0.19–0.89)	0.024	0.22 (0.08–0.59)	0.003
Community-acquired bacteremia	0.34 (0.13–0.86)	0.023	1.16 (0.31–4.34)	0.831
Liver abscess as a source of infection	0.21 (0.05–0.93)	0.039	0.49 (0.10–2.45)	0.382
Malignancy	2.92 (1.35–6.31)	0.007	3.09 (0.92–10.42)	0.068
Chronic kidney disease	2.16 (1.01–4.66)	0.048	1.48 (0.48–4.63)	0.498
Chronic obstructive lung disease	4.19 (1.15–15.26)	0.030	6.27 (1.12–34.97)	0.036
Immunosuppression	4.16 (1.82–9.53)	0.001	2.77 (0.90–8.49)	0.075
Charlson’s comorbidity index	1.20 (1.07–1.35)	0.002	1.02 (0.86–1.21)	0.846
Nasogastric/nasojejunal tube at onset of bacteremia	3.07 (1.39–6.80)	0.006	1.15 (0.31–4.32)	0.832
Urinary catheter at onset of bacteremia	2.69 (1.24–5.86)	0.013	2.38 (0.69–8.24)	0.172
Prior antibiotic exposure	4.07 (1.87–8.88)	<0.001	2.10 (0.64–6.89)	0.221
APACHE II score	1.10 (1.05–1.15)	<0.001	1.07 (1.01–1.13)	0.033

^*a*^ APACHE, Acute Physiology and Chronic Health Evaluation.

### Subgroup analyses of critically ill patients with hvKp bacteremia

Among the 90 patients with an APACHE II score of 15 or higher, the 14-day and 28-day mortality rates were 13.3% (*n* = 12) and 23.3% (*n* = 21), respectively. Carbapenems were used in 27 patients (30.0%). Table S4 showed the comparison of clinical characteristics in hvKp bacteremia patients with an APACHE II score of 15 or higher receiving non-carbapenems versus carbapenems as definitive antibiotics. Patients in the carbapenem group had a higher disease severity than those in the non-carbapenem group, as indicated by the median Pitt bacteremia score (4.0 versus 3.0, *P* = 0.039) and median APACHE II score (23.0 versus 19.0, *P* = 0.001). There is a statistically nonsignificant trend toward a higher 28-day mortality in the carbapenem group than in the non-carbapenem group (33.3% versus 19.0%, *P* = 0.142) (Table S5). In the multivariate logistic regression model, chronic obstructive lung disease was the independent risk factor for 28-day mortality (OR, 15.04; 95% CI, 1.50 to 150.37; *P* = 0.021). Definitive treatment with carbapenems for hvKp bacteremia was not associated with 28-day mortality (OR, 2.63; 95% CI, 0.58 to 12.00; *P* = 0.212) (Table 4).

**TABLE 4:** Multivariate logistic regression analysis of predictors for 28-day mortality in patients with hypervirulent *K. pneumoniae* bacteremia with an APACHE II score of 15 or higher^*a*^

Variables	Univariate analysis OR (95% CI)	*P* value	Multivariate analysis OR (95% CI)	*P* value
Definitive antibiotic				
Non-carbapenems	1.00 (reference)		1.00 (reference)	
Carbapenems	2.13 (0.77–5.88)	0.147	2.63 (0.58–12.00)	0.212
Male	0.25 (0.09–0.68)	0.007	0.13 (0.04–0.49)	0.002
Malignancy	3.12 (1.13–8.57)	0.028	2.31 (0.61–8.79)	0.221
Chronic obstructive lung disease	7.88 (1.33–46.69)	0.023	15.04 (1.50–150.37)	0.021
Immunosuppression	3.25 (1.09–9.66)	0.035	0.92 (0.21–3.98)	0.908
Prior antibiotic exposure	4.00 (1.42–11.27)	0.009	1.91 (0.43–8.54)	0.397
Source control	0.11 (0.01–0.85)	0.034	0.12 (0.01–1.18)	0.069

^*a*^ APACHE, Acute Physiology and Chronic Health Evaluation.

### Subgroup analyses of patients infected with hvKp strains that exhibited wild-type antibiotic susceptibility

Among the 195 patients infected with hvKp strains that exhibited wild-type antibiotic susceptibility, the 14-day and 28-day mortality rates were 8.7% (*n* = 17) and 12.8% (*n* = 25), respectively. We found 34 patients (17.4%) who still received carbapenems as definitive therapy. Table S6 showed the comparison of clinical characteristics in these patients receiving non-carbapenems versus carbapenems as definitive antibiotics. The 28-day mortality in the carbapenem group was higher than that of the non-carbapenem group (23.5% versus 10.6%, *P* = 0.040) (Table S7). In the multivariate logistic regression model, APACHE II score was the independent risk factor for 28-day mortality (OR, 1.10; 95% CI, 1.03 to 1.18; *P* = 0.008). Definitive treatment with carbapenems for hvKp bacteremia was not associated with 28-day mortality (OR, 1.29; 95% CI, 0.39 to 4.26; *P* = 0.676) (Table 5).

**TABLE 5:** Multivariate logistic regression analysis of predictors for 28-day mortality in patients infected with hypervirulent *K. pneumoniae* strains that exhibited wild-type antibiotic susceptibility^*a*^

Variables	Univariate analysis OR (95% CI)	*P* value	Multivariate analysis OR (95% CI)	*P* value
Definitive antibiotic				
Non-carbapenems	1.00 (reference)		1.00 (reference)	
Carbapenems	2.61 (1.02–6.67)	0.045	1.29 (0.39–4.26)	0.676
Male	0.35 (0.15–0.81)	0.015	0.22 (0.08–0.61)	0.004
Community-acquired bacteremia	0.36 (0.14–0.96)	0.040	0.82 (0.22–3.05)	0.767
Immunosuppression	3.99 (1.57–10.17)	0.004	3.10 (0.85–11.32)	0.087
Charlson’s comorbidity index	1.21 (1.04–1.41)	0.015	1.09 (0.90–1.32)	0.384
Nasogastric/nasojejunal tube at onset of bacteremia	3.69 (1.50–9.11)	0.005	1.65 (0.36–7.64)	0.522
Urinary catheter at onset of bacteremia	2.93 (1.22–6.99)	0.016	1.23 (0.28–5.32)	0.783
Prior antibiotic exposure	4.31 (1.79–10.37)	0.001	3.05 (0.87–10.73)	0.082
APACHE II score	1.12 (1.06–1.18)	<0.001	1.10 (1.03–1.18)	0.008

^*a*^ APACHE, Acute Physiology and Chronic Health Evaluation.

### Risk factors for 28-day mortality in patients with hvKp bacteremia using the propensity score-matched analysis

We further used propensity score-matched analysis to study risk factors for 28-day mortality in overall patients with hvKp bacteremia, critically ill patients with hvKp bacteremia, and patients infected with wild-type hvKp strains. The clinical characteristics of the propensity score-matched cohorts were detailed in Tables 1 and 2 for overall patients with hvKp bacteremia, in Tables S4 and S5 for critically ill patients with hvKp bacteremia, and in Tables S6 and S7 for patients infected with wild-type hvKp strains. Definitive carbapenem therapy was analyzed in the multivariate model regardless of its significance in the univariate analysis and was not associated with 28-day mortality across three propensity score-matched populations (Table S8 through S10).

## DISCUSSION

In this study, we found that around one-fourth of patients received carbapenems as definitive therapy for hvKp bacteremia. Definitive therapy with carbapenems for hvKp bacteremia was not associated with 28-day mortality. The results were consistent throughout the analyses for critically ill patients and those infected with hvKp strains exhibiting wild-type antibiotic susceptibility.

There is currently no consensus about the treatment recommendation for hvKp bacteremia (2). The detailed information about the treatment of hvKp bacteremia in the literature was limited. The use of carbapenem was common in clinical practice. In Brazil, de Oliveira Franco et al. reported a 57-year-old diabetic lady with invasive liver abscess syndrome with meningitis and a spinal abscess caused by hvKp strain with wild-type susceptibility (10). In China, Wu et al. described a 72-year-old lady with liver cirrhosis who had hvKp bacteremia with wild-type susceptibility and gas gangrene of lower limbs (9). Two of the aforementioned patients were treated with about an 8-week meropenem (9, 10). In Japan, Namikawa et al. retrospectively reviewed 24 patients with hvKp bacteremia (8). The hvKp strains in the study were only resistant to ampicillin, ampicillin/sulbactam, and trimethoprim/sulfamethoxazole, but carbapenems were the most commonly used regimen (25%) (8). It is apparent that definitive carbapenem treatment in real-world practice was not uncommon, despite the absence of MDR phenotype in these hvKp strains.

The comparison of the effectiveness of different antibiotics in hvKp bacteremia was lacking in the literature. In Taiwan, we previously showed that fluoroquinolones demonstrated comparable clinical efficacy to β-lactams as the major treatment for liver abscess due to hvKp strains, even in critically ill patients, with 43.2% experiencing bacteremia (7). In the current study, we first found that use of carbapenems as definitive therapy for hvKp bacteremia was not independently associated with a decreased 28-day mortality, even in critically ill patients. The unfamiliarity with the hyKp bacteremia and fear of the severe disease led to the overuse of carbapenem, and our findings demonstrated a clear guide for antimicrobial stewardship.

We found that most (82.6%) hvKp strains exhibited wild-type susceptibility (susceptible to various antibiotics, with resistance only to ampicillin), reflecting the microbiological nature of hvKp strains (2). We also identified 11.9% (*n* = 28) of *K. pneumoniae* strains that were MDR-hvKp strains in the current study. It is reasonable that the proportion of MDR-hvKp strains was higher in the carbapenem group because of the tendency of carbapenem use in strains with an MDR phenotype. However, we found near one-fifth of patients infected with hvKp strains exhibiting wild-type antibiotic susceptibility still received carbapenems as definitive therapy, but the therapy with carbapenem was not associated with a survival benefit. The findings highlighted that a carbapenem-sparing strategy is appropriate in hvKp bacteremia, and we recommended that physicians use antibiotics according to the antimicrobial susceptibility tests.

Our study is the first one to compare the outcomes of patients with hvKp bacteremia receiving carbapenem versus non-carbapenem treatment. We have included the largest sample size (236 patients) regarding this issue and concluded that carbapenem was unnecessary in these patients. Our study had several limitations. First, our study was performed in a single tertiary-care teaching hospital where patient demographics, clinical practices, and treatment outcomes may vary from those in other non-tertiary-care hospitals. Second, this was an observational study because it is difficult to design and enroll patients for randomized clinical trials. The gold standard for inferring associations between treatment choice and outcome in observational studies is likely to be target trial emulation. We did not use target trial emulation; however, we attempted to adjust the differences between patients given carbapenems and non-carbapenems using multivariable regression and propensity score-matched analyses. Although we included probable confounders and prognostic factors related to mortality, there might still be some degree of unmeasured confounding present. Third, the study cohort was from 2015 to 2017, and resistance to cephalosporins may have increased since then. Last, we did not collect information on the resistance development or adverse events between the two groups, as our study was not specifically designed to investigate these aspects.

In summary, definitive therapy with carbapenems for patients with hvKp bacteremia was not better than non-carbapenem therapy, even in critically ill patients. Our findings will provide insight into antimicrobial stewardship in the endemic region of hvKp infections.

## SUPPLEMENTAL MATERIAL

Supplemental tables aac.00846-25-s0001.docx Tables S1 to S10.

ASM does not own the copyrights to Supplemental Material that may be linked to, or accessed through, an article. The authors have granted ASM a non-exclusive, world-wide license to publish the Supplemental Material files. Please contact the corresponding author directly for reuse.
