# Supplementary material for: Carbapenems versus non-carbapenems as definitive treatment for hypervirulent Klebsiella pneumoniae bacteremia in Taiwan
Source: Antimicrob Agents Chemother. 2025 Nov 18;69(12):e00846-25. doi: 10.1128/aac.00846-25 (PMC12691612; doi:10.1128/aac.00846-25)
Supplement: Supplemental tables — Tables S1 to S10. [file aac.00846-25-s0001.docx]

**Table S1.** Antimicrobial resistance rates of clinical isolates of patients with hypervirulent *K. pneumoniae* bacteremia in the non-carbapenems versus carbapenems group

| Variable | Non-carbapenems (n=184) | Carbapenems (n=52) | *p* value |
| --- | --- | --- | --- |
| Amikacin | 0 (0.0) | 2 (3.8) | 0.048 |
| Gentamicin | 1 (0.5) | 8 (15.4) | <0.001 |
| Cefuroxime | 15 (8.2) | 17 (32.7) | <0.001 |
| Ceftriaxone | 2 (1.1) | 14 (26.9) | <0.001 |
| Cefepime | 0 (0.0) | 5 (9.6) | <0.001 |
| Ciprofloxacin | 2 (1.1) | 10 (19.2) | <0.001 |
| Levofloxacin | 2 (1.1) | 9 (17.3) | <0.001 |
| Ertapenem | 0 (0.0) | 7 (13.5) | <0.001 |
| Imipenem | 0 (0.0) | 1 (1.9) | 0.220 |
| Tigecycline | 2 (1.1) | 2 (3.8) | 0.212 |
| Trimethoprim-sulfamethoxazole | 5 (2.7) | 13 (25.0) | <0.001 |
| Wild-type antibiotic susceptibility^a^ | 161 (87.5) | 34 (65.4) | <0.001 |
| Multidrug resistance^b^ | 11 (6.0) | 17 (32.7) | <0.001 |

Data are presented as number (%) of isolates resistant to the antibiotic indicated, unless stated otherwise

^a^Wild-type antibiotic susceptibility was defined in the isolates as susceptibility to all antibiotics except for ampicillin

^b^Multidrug resistance was defined in the isolates as nonsusceptibility to at least one agent in three or more antimicrobial categories

**Table S2.** Comparison of microbiological characteristic among clinical isolates of patients with hypervirulent *K. pneumoniae* bacteremia in the non-carbapenems versus carbapenems group

| Variable | Non-carbapenems (n=184) | Carbapenems (n=52) | *p* value |
| --- | --- | --- | --- |
| Capsular type K1 | 52 (28.3) | 12 (23.1) | 0.458 |
| Capsular type K2 | 48 (26.1) | 12 (23.1) | 0.660 |
| Capsular type K1 and K2 | 100 (54.3) | 24 (46.2) | 0.296 |
| Capsular type K1, K2, K5, K20, K54, and K57 | 148 (80.4) | 35 (67.3) | 0.045 |
| Presence of plasmid *rmpA* | 176 (95.7) | 49 (94.2) | 0.710 |
| Presence of plasmid *rmpA2* | 172 (93.5) | 46 (88.5) | 0.229 |

**Table S3**. Comparison of characteristics in patients with hypervirulent *K. pneumoniae* bacteremia by survival status within 28 days

|  | Survivors (n=205) | Non-survivors (n=31) | *p* value |
| --- | --- | --- | --- |
| Demographics |  |  |  |
| Age, median (IQR), years | 71.0 (60.0-81.0) | 78.0 (63.0-82.5) | 0.238 |
| Gender, male | 142 (69.3) | 15 (48.4) | 0.022 |
| Location of infection acquisition |  |  |  |
| Community | 85 (41.5) | 6 (19.4) | 0.018 |
| Healthcare associated | 60 (29.3) | 12 (38.7) | 0.287 |
| Hospital | 60 (29.3) | 13 (41.9) | 0.155 |
| Source of infection |  |  |  |
| Respiratory system | 41 (20.0) | 11 (35.5) | 0.053 |
| Urinary | 46 (22.4) | 3 (9.7) | 0.152 |
| Intra-abdominal^a^ | 36 (17.6) | 9 (29.0) | 0.130 |
| Liver abscess | 50 (24.4) | 2 (6.5) | 0.034 |
| Primary bacteremia | 30 (14.6) | 5 (16.1) | 0.827 |
| Skin and soft tissue | 7 (3.4) | 2 (6.5) | 0.336 |
| Intravenous catheter | 0 (0.0) | 0 (0.0) | N/A |
| Others | 3 (1.5) | 1 (3.2) | 0.433 |
| Disseminated infections | 8 (3.9) | 2 (6.5) | 0.624 |
| Underlying diseases |  |  |  |
| Malignancy | 66 (32.2) | 18 (58.1) | 0.005 |
| Diabetes mellitus | 94 (45.9) | 12 (38.7) | 0.456 |
| Chronic kidney disease | 80 (39.0) | 18 (58.1) | 0.045 |
| Hemodialysis | 17 (8.3) | 1 (3.2) | 0.481 |
| Congestive heart failure | 17 (8.3) | 3 (9.7) | 0.733 |
| Liver cirrhosis | 28 (13.7) | 4 (12.9) | >0.999 |
| Cerebral vascular disease | 24 (11.7) | 1 (3.2) | 0.215 |
| Chronic obstructive lung disease | 7 (3.4) | 4 (12.9) | 0.042 |
| Collagen vascular disease | 6 (2.9) | 2 (6.5) | 0.283 |
| Transplantation | 1 (0.5) | 0 (0.0) | >0.999 |
| Immunosuppression^b^ | 27 (13.2) | 12 (38.7) | <0.001 |
| Charlson’s comorbidity index, median (IQR) | 6.0 (4.0-8.0) | 7.0 (6.0-9.0) | 0.002 |
| Invasive procedures and devices at onset of bacteremia |  |  |  |
| Central venous catheter | 27 (13.2) | 6 (19.4) | 0.355 |
| Nasogastric/Nasojejunal tube | 39 (19.0) | 13 (41.9) | 0.004 |
| Urinary catheter | 48 (23.4) | 14 (45.2) | 0.010 |
| Endotracheal tube^c^ | 19 (9.3) | 4 (12.9) | 0.517 |
| Tracheostomy | 8 (3.9) | 2 (6.5) | 0.624 |
| Surgical drainage | 17 (8.3) | 4 (12.9) | 0.494 |
| Surgery within 2 weeks | 29 (14.1) | 4 (12.9) | >0.999 |
| Prior antibiotic exposure |  |  |  |
| Any antibiotic | 52 (25.4) | 18 (58.1) | <0.001 |
| 1^st^ or 2^nd^ generation cephalosporin^d^ | 30 (14.6) | 10 (32.3) | 0.015 |
| 3^rd^ or 4^th^ generation cephalosporin^e^ | 10 (4.9) | 3 (9.7) | 0.387 |
| β-lactam and β-lactamase inhibitor^f^ | 16 (7.8) | 9 (29.0) | <0.001 |
| Carbapenem^g^ | 14 (6.8) | 1 (3.2) | 0.700 |
| Fluoroquinolone^h^ | 8 (3.9) | 3 (9.7) | 0.163 |
| Aminoglycoside^i^ | 2 (1.0) | 1 (3.2) | 0.346 |
| Tigecycline | 4 (2.0) | 2 (6.5) | 0.179 |
| Glycopeptide^j^ | 7 (3.4) | 3 (9.7) | 0.130 |
| Metronidazole | 10 (4.9) | 0 (0.0) | 0.367 |
| Source control | 59 (28.8) | 5 (16.1) | 0.140 |
| Requirement of ICU admission | 43 (21.0) | 7 (22.6) | 0.838 |
| Pitt bacteremia score, median (IQR) | 1.0 (0.0-3.0) | 3.0 (0.5-4.0) | 0.078 |
| APACHE II score, median (IQR) | 11.0 (7.0-17.0) | 18.0 (12.0-23.5) | <0.001 |
| Appropriate empirical antimicrobial therapy | 191 (93.2) | 29 (93.5) | >0.999 |
| Carbapenem as definitive therapy | 40 (19.5) | 12 (38.7) | 0.016 |
| Septic shock when bacteremia | 61 (29.8) | 13 (41.9) | 0.173 |
| Respiratory failure requiring mechanical ventilation | 19 (9.3) | 4 (12.9) | 0.517 |

Data are presented as number (%) of patients, unless stated otherwise.

IQR, interquartile range; APACHE, Acute Physiology And Chronic Health Evaluation; N/A, not applicable

^a^Intra-abdominal infection was defined as infections of single organs of abdomen with or without extension into the peritoneal space with exclusion of liver abscess

^b^Immunosuppression was defined as meeting one of the following criteria: neutropenia, use of corticosteroids, or receiving chemotherapy.

^c^Endotracheal tube was defined as patient being intubated at the onset of bacteremia.

^d^Including cefazolin and cefuroxime

^e^Including cefoperazone, ceftriaxone, cefotaxime, cefepime, and cefpirome

^f^Including amoxicillin/clavulanate, ampicillin/sulbactam, piperacillin/tazobactam, and ticarcillin/clavulanate

^g^Including ertapenem, imipenem, meropenem, and doripenem

^h^Including ciprofloxacin, levofloxacin, and moxifloxacin

^i^Including amikacin, gentamicin and isepamicin.

^j^Including vancomycin and teicoplanin

**Table S4**. Comparison of clinical characteristics in patients with hypervirulent *K. pneumoniae* bacteremia with an APACHE II score of 15 or higher receiving non-carbapenems versus carbapenems as definitive antibiotic

|  | Original cohort | | |  | Propensity score-matched cohort | | |
| --- | --- | --- | --- | --- | --- | --- | --- |
|  | Non-carbapenems (n=63) | Carbapenems (n=27) | *p* value |  | Non-carbapenems (n=54) | Carbapenems (n=27) | *p* value |
| Demographics |  |  |  |  |  |  |  |
| Age, median (IQR), years | 78.0 (64.0-84.5) | 75.0 (67.0-80.0) | 0.332 |  | 78.0 (64.0-84.8) | 75.0 (67.0-80.0) | 0.261 |
| Gender, male | 44 (69.8) | 17 (63.0) | 0.522 |  | 37 (68.5) | 17 (63.0) | 0.803 |
| Location of infection acquisition |  |  |  |  |  |  |  |
| Community | 24 (38.1) | 5 (18.5) | 0.087 |  | 19 (35.2) | 5 (18.5) | 0.197 |
| Healthcare associated | 21 (33.3) | 7 (25.9) | 0.487 |  | 19 (35.2) | 7 (25.9) | 0.556 |
| Hospital | 18 (28.6) | 15 (55.6) | 0.015 |  | 16 (29.6) | 15 (55.6) | 0.043 |
| Source of infection |  |  |  |  |  |  |  |
| Respiratory system | 21 (33.3) | 11 (40.7) | 0.501 |  | 21 (38.9) | 11 (40.7) | >0.999 |
| Urinary | 9 (14.3) | 3 (11.1) | >0.999 |  | 8 (14.8) | 3 (11.1) | 0.909 |
| Intra-abdominal^a^ | 11 (17.5) | 1 (3.7) | 0.099 |  | 6 (11.1) | 1 (3.7) | 0.485 |
| Liver abscess | 11 (17.5) | 6 (22.2) | 0.597 |  | 11 (20.4) | 6 (22.2) | >0.999 |
| Primary bacteremia | 7 (11.1) | 6 (22.2) | 0.169 |  | 5 (9.3) | 6 (22.2) | 0.207 |
| Skin and soft tissue | 5 (7.9) | 1 (3.7) | 0.664 |  | 4 (7.4) | 1 (3.7) | 0.870 |
| Intravenous catheter | 0 (0.0) | 0 (0.0) | N/A |  | 0 (0.0) | 0 (0.0) | N/A |
| Others | 1 (1.6) | 1 (3.7) | 0.512 |  | 1 (1.9) | 1 (3.7) | >0.999 |
| Disseminated infection | 2 (3.2) | 2 (7.4) | 0.580 |  | 2 (3.7) | 2 (7.4) | 0.856 |
| Underlying diseases |  |  |  |  |  |  |  |
| Malignancy | 21 (33.3) | 8 (29.6) | 0.730 |  | 18 (33.3) | 8 (29.6) | 0.933 |
| Diabetes mellitus | 28 (44.4) | 17 (63.0) | 0.107 |  | 26 (48.1) | 17 (63.0) | 0.306 |
| Chronic kidney disease | 28 (44.4) | 19 (70.4) | 0.024 |  | 26 (48.1) | 19 (70.4) | 0.097 |
| Hemodialysis | 6 (9.5) | 5 (18.5) | 0.233 |  | 6 (11.1) | 5 (18.5) | 0.566 |
| Congestive heart failure | 6 (9.5) | 5 (18.5) | 0.233 |  | 5 (9.3) | 5 (18.5) | 0.403 |
| Liver cirrhosis | 8 (12.7) | 1 (3.7) | 0.269 |  | 7 (13.0) | 1 (3.7) | 0.357 |
| Cerebral vascular disease | 10 (15.9) | 5 (18.5) | 0.758 |  | 8 (14.8) | 5 (18.5) | 0.915 |
| Chronic obstructive lung disease | 6 (9.5) | 0 (0.0) | 0.173 |  | 6 (11.1) | 0 (0.0) | 0.177 |
| Collagen vascular disease | 3 (4.8) | 0 (0.0) | 0.551 |  | 3 (5.6) | 0 (0.0) | 0.533 |
| Transplantation | 0 (0.0) | 0 (0.0) | N/A |  | 0 (0.0) | 0 (0.0) | N/A |
| Immunosuppression^b^ | 12 (19.0) | 7 (25.9) | 0.464 |  | 11 (20.4) | 7 (25.9) | 0.777 |
| Charlson’s comorbidity index, median (IQR) | 7.0 (5.0-9.0) | 6.0 (5.0-8.5) | 0.338 |  | 7.0 (5.0-9.0) | 6.0 (5.0-8.5) | 0.239 |
| Invasive procedures and devices at onset of bacteremia |  |  |  |  |  |  |  |
| Central venous catheter | 10 (15.9) | 13 (48.1) | 0.001 |  | 9 (16.7) | 13 (48.1) | 0.006 |
| Nasogastric/Nasojejunal tube | 24 (38.1) | 12 (44.4) | 0.573 |  | 21 (38.9) | 12 (44.4) | 0.810 |
| Urinary catheter | 20 (31.7) | 13 (48.1) | 0.139 |  | 18 (33.3) | 13 (48.1) | 0.293 |
| Endotracheal tube^c^ | 12 (19.0) | 7 (25.9) | 0.464 |  | 11 (20.4) | 7 (25.9) | 0.777 |
| Tracheostomy | 3 (4.8) | 5 (18.5) | 0.050 |  | 3 (5.6) | 5 (18.5) | 0.148 |
| Surgical drainage | 5 (7.9) | 5 (18.5) | 0.143 |  | 4 (7.4) | 5 (18.5) | 0.261 |
| Surgery within 2 weeks | 8 (12.7) | 5 (18.5) | 0.472 |  | 7 (13.0) | 5 (18.5) | 0.740 |
| Prior antibiotic exposure |  |  |  |  |  |  |  |
| Any antibiotic | 18 (28.6) | 19 (70.4) | <0.001 |  | 17 (31.5) | 19 (70.4) | 0.002 |
| 1^st^ or 2^nd^ generation cephalosporin^d^ | 10 (15.9) | 6 (22.2) | 0.470 |  | 10 (18.5) | 6 (22.2) | 0.921 |
| 3^rd^ or 4^th^ generation cephalosporin^e^ | 2 (3.2) | 4 (14.8) | 0.064 |  | 2 (3.7) | 4 (14.8) | 0.177 |
| β-lactam and β-lactamase inhibitor^f^ | 10 (15.9) | 10 (37.0) | 0.027 |  | 9 (16.7) | 10 (37.0) | 0.078 |
| Carbapenem^g^ | 2 (3.2) | 4 (14.8) | 0.064 |  | 2 (3.7) | 4 (14.8) | 0.177 |
| Fluoroquinolone^h^ | 2 (3.2) | 4 (14.8) | 0.064 |  | 2 (3.7) | 4 (14.8) | 0.177 |
| Aminoglycoside^i^ | 0 (0.0) | 2 (7.4) | 0.088 |  | 0 (0.0) | 2 (7.4) | 0.206 |
| Tigecycline | 0 (0.0) | 5 (18.5) | 0.002 |  | 0 (0.0) | 5 (18.5) | 0.006 |
| Glycopeptide^j^ | 2 (3.2) | 3 (11.1) | 0.157 |  | 2 (3.7) | 3 (11.1) | 0.414 |
| Metronidazole | 2 (3.2) | 1 (3.7) | >0.999 |  | 2 (3.7) | 1 (3.7) | >0.999 |
| Source control | 17 (27.0) | 6 (22.2) | 0.635 |  | 12 (22.2) | 6 (22.2) | >0.999 |
| Septic shock when bacteremia | 31 (49.2) | 20 (74.1) | 0.029 |  | 27 (50.0) | 20 (74.1) | 0.067 |
| Respiratory failure requiring mechanical ventilation | 12 (19.0) | 5 (18.5) | 0.464 |  | 12 (22.2) | 5 (18.5) | 0.923 |
| Pitt bacteremia score, median (IQR) | 3.0 (1.0-4.0) | 4.0 (2.0-6.0) | 0.039 |  | 3.0 (1.0-4.0) | 4.0 (2.0-6.0) | 0.054 |
| APACHE II score, median (IQR) | 19.0 (17.0-22.0) | 23.0 (19.0-28.5) | 0.001 |  | 19.0 (17.0-22.0) | 23.0 (19.0-28.5) | 0.002 |

Data are presented as number (%) of patients, unless stated otherwise.

IQR, interquartile range; APACHE, Acute Physiology And Chronic Health Evaluation; N/A, not applicable

^a^Intra-abdominal infection was defined as infections of single organs of abdomen with or without extension into the peritoneal space with exclusion of liver abscess

^b^Immunosuppression was defined as meeting one of the following criteria: neutropenia, use of corticosteroids, or receiving chemotherapy.

^c^Endotracheal tube was defined as patient being intubated at the onset of bacteremia.

^d^Including cefazolin and cefuroxime

^e^Including cefoperazone, ceftriaxone, cefotaxime, cefepime, and cefpirome

^f^Including amoxicillin/clavulanate, ampicillin/sulbactam, piperacillin/tazobactam, and ticarcillin/clavulanate

^g^Including ertapenem, imipenem, meropenem, and doripenem

^h^Including ciprofloxacin, levofloxacin, and moxifloxacin

^i^Including amikacin, gentamicin and isepamicin.

^j^Including vancomycin and teicoplanin

**Table S5**. Comparison of clinical outcomes in patients with hypervirulent *K. pneumoniae* bacteremia with an APACHE II score of 15 or higher receiving non-carbapenems versus carbapenems as definitive antibiotic

|  | Original cohort | | |  | Propensity score-matched cohort | | |
| --- | --- | --- | --- | --- | --- | --- | --- |
|  | Non-carbapenems (n=63) | Carbapenems (n=27) | *p* value |  | Non-carbapenems (n=54) | Carbapenems (n=27) | *p* value |
| Appropriate empirical antimicrobial therapy | 62 (98.4) | 21 (77.8) | 0.003 |  | 53 (98.1) | 21 (77.8) | 0.008 |
| Length of hospital stay, median (IQR), days | 22.0 (16.0-45.0) | 40.0 (20.5-52.0) | 0.104 |  | 22.0  (15.3-43.5) | 40.0  (20.5-52.0) | 0.079 |
| 14-day mortality | 7 (11.1) | 5 (18.5) | 0.343 |  | 7 (13.0) | 5 (18.5) | 0.740 |
| 28-day mortality | 12 (19.0) | 9 (33.3) | 0.142 |  | 11 (20.4) | 9 (33.3) | 0.316 |
| In-hospital mortality | 17 (27.0) | 13 (48.1) | 0.051 |  | 14 (25.9) | 13 (48.1) | 0.080 |

Data are presented as number (%) of patients, unless stated otherwise.

IQR, interquartile range

**Table S6**. Comparison of clinical characteristics in patients infected with hypervirulent *K. pneumoniae* strains that exhibited wild-type antibiotic susceptibility receiving non-carbapenems versus carbapenems as definitive antibiotic

|  | Original cohort | | |  | Propensity score-matched cohort | | |
| --- | --- | --- | --- | --- | --- | --- | --- |
|  | Non-carbapenems (n=161) | Carbapenems (n=34) | *p* value |  | Non-carbapenems (n=68) | Carbapenems (n=34) | *p* value |
| Demographics |  |  |  |  |  |  |  |
| Age, median (IQR), years | 72.0 (59.0-83.0) | 75.5 (62.0-80.0) | 0.322 |  | 76.0 (63.0-84.3) | 75.5 (62.5-80.0) | 0.812 |
| Gender, male | 108 (67.1) | 21 (61.8) | 0.552 |  | 40 (58.8) | 21 (61.8) | 0.943 |
| Location of infection acquisition |  |  |  |  |  |  |  |
| Community | 73 (45.3) | 12 (35.3) | 0.283 |  | 31 (45.6) | 12 (35.3) | 0.436 |
| Healthcare associated | 53 (32.9) | 9 (26.5) | 0.463 |  | 19 (27.9) | 9 (26.5) | >0.999 |
| Hospital | 35 (21.7) | 13 (38.2) | 0.042 |  | 18 (26.5) | 13 (38.2) | 0.322 |
| Source of infection |  |  |  |  |  |  |  |
| Respiratory system | 32 (19.9) | 12 (35.3) | 0.051 |  | 16 (23.5) | 12 (35.3) | 0.308 |
| Urinary | 35 (21.7) | 4 (11.8) | 0.241 |  | 13 (19.1) | 4 (11.8) | 0.511 |
| Intra-abdominal^a^ | 28 (17.4) | 5 (14.7) | 0.704 |  | 9 (13.2) | 5 (14.7) | >0.999 |
| Liver abscess | 41 (25.5) | 7 (20.6) | 0.549 |  | 16 (23.5) | 7 (20.6) | 0.933 |
| Primary bacteremia | 22 (13.7) | 6 (17.6) | 0.547 |  | 12 (17.6) | 6 (17.6) | >0.999 |
| Skin and soft tissue | 8 (5.0) | 1 (2.9) | >0.999 |  | 4 (5.9) | 1 (2.9) | 0.871 |
| Intravenous catheter | 0 (0.0) | 0 (0.0) | N/A |  | 0 (0.0) | 0 (0.0) | N/A |
| Others | 4 (2.5) | 1 (2.9) | 0.878 |  | 1 (1.5) | 1 (2.9) | >0.999 |
| Disseminated infection | 7 (4.3) | 2 (5.9) | 0.658 |  | 3 (4.4) | 2 (5.9) | >0.999 |
| Underlying diseases |  |  |  |  |  |  |  |
| Malignancy | 59 (36.6) | 10 (29.4) | 0.423 |  | 25 (36.8) | 10 (29.4) | 0.606 |
| Diabetes mellitus | 68 (42.2) | 19 (55.9) | 0.146 |  | 35 (51.5) | 19 (55.9) | 0.833 |
| Chronic kidney disease | 61 (37.9) | 19 (55.9) | 0.053 |  | 30 (44.1) | 19 (55.9) | 0.362 |
| Hemodialysis | 8 (5.0) | 2 (5.9) | 0.687 |  | 3 (4.4) | 2 (5.9) | >0.999 |
| Congestive heart failure | 8 (5.0) | 6 (17.6) | 0.009 |  | 6 (8.8) | 6 (17.6) | 0.328 |
| Liver cirrhosis | 27 (16.8) | 2 (5.9) | 0.119 |  | 9 (13.2) | 2 (5.9) | 0.430 |
| Cerebral vascular disease | 15 (9.3) | 3 (8.8) | >0.999 |  | 8 (11.8) | 3 (8.8) | 0.910 |
| Chronic obstructive lung disease | 8 (5.0) | 2 (5.9) | 0.687 |  | 5 (7.4) | 2 (5.9) | >0.999 |
| Collagen vascular disease | 7 (4.3) | 0 (0.0) | 0.608 |  | 0 (0.0) | 0 (0.0) | N/A |
| Transplantation | 0 (0.0) | 1 (2.9) | 0.174 |  | 0 (0.0) | 1 (2.9) | 0.722 |
| Immunosuppression^b^ | 22 (13.7) | 8 (23.5) | 0.147 |  | 15 (22.1) | 8 (23.5) | >0.999 |
| Charlson’s comorbidity index, median (IQR) | 6.0 (4.0-8.0) | 6.0 (5.0-9.0) | 0.550 |  | 7.0 (5.0-9.0) | 6.0 (5.0-9.0) | 0.589 |
| Invasive procedures and devices at onset of bacteremia |  |  |  |  |  |  |  |
| Central venous catheter | 14 (8.7) | 9 (26.5) | 0.004 |  | 7 (10.3) | 9 (26.5) | 0.067 |
| Nasogastric/Nasojejunal tube | 26 (16.1) | 10 (29.4) | 0.070 |  | 10 (14.7) | 10 (29.4) | 0.134 |
| Urinary catheter | 34 (21.1) | 13 (38.2) | 0.034 |  | 16 (23.5) | 13 (38.2) | 0.187 |
| Endotracheal tube^c^ | 13 (8.1) | 3 (8.8) | >0.999 |  | 3 (4.4) | 3 (8.8) | 0.655 |
| Tracheostomy | 1 (0.6) | 2 (5.9) | 0.079 |  | 1 (1.5) | 2 (5.9) | 0.534 |
| Surgical drainage | 10 (6.2) | 3 (8.8) | 0.703 |  | 4 (5.9) | 3 (8.8) | 0.890 |
| Surgery within 2 weeks | 16 (9.9) | 8 (23.5) | 0.028 |  | 9 (13.2) | 8 (23.5) | 0.301 |
| Prior antibiotic exposure |  |  |  |  |  |  |  |
| Any antibiotic | 30 (18.6) | 12 (35.3) | 0.032 |  | 13 (19.1) | 12 (35.3) | 0.122 |
| 1^st^ or 2^nd^ generation cephalosporin^d^ | 21 (13.0) | 6 (17.6) | 0.480 |  | 6 (8.8) | 6 (17.6) | 0.328 |
| 3^rd^ or 4^th^ generation cephalosporin^e^ | 7 (4.3) | 1 (2.9) | >0.999 |  | 1 (1.5) | 1 (2.9) | >0.999 |
| β-lactam and β-lactamase inhibitor^f^ | 8 (5.0) | 7 (20.6) | 0.002 |  | 6 (8.8) | 7 (20.6) | 0.172 |
| Carbapenem^g^ | 4 (2.5) | 1 (2.9) | >0.999 |  | 2 (2.9) | 1 (2.9) | >0.999 |
| Fluoroquinolone^h^ | 1 (0.6) | 3 (8.8) | 0.017 |  | 1 (1.5) | 3 (8.8) | 0.207 |
| Aminoglycoside^i^ | 0 (0.0) | 0 (0.0) | N/A |  | 0 (0.0) | 0 (0.0) | N/A |
| Tigecycline | 0 (0.0) | 1 (2.9) | 0.174 |  | 0 (0.0) | 1 (2.9) | 0.722 |
| Glycopeptide^j^ | 2 (1.2) | 2 (5.9) | 0.141 |  | 0 (0.0) | 2 (5.9) | 0.207 |
| Metronidazole | 4 (2.5) | 2 (5.9) | 0.281 |  | 3 (4.4) | 2 (5.9) | >0.999 |
| Source control | 44 (27.3) | 12 (35.3) | 0.351 |  | 25 (36.8) | 12 (35.3) | >0.999 |
| Septic shock when bacteremia | 44 (27.3) | 14 (41.2) | 0.109 |  | 23 (33.8) | 14 (41.2) | 0.610 |
| Respiratory failure requiring mechanical ventilation | 20 (12.4) | 5 (14.7) | 0.717 |  | 7 (10.3) | 5 (14.7) | 0.744 |
| Requirement of ICU admission | 36 (22.4) | 10 (29.4) | 0.379 |  | 19 (27.9) | 10 (29.4) | >0.999 |
| Pitt bacteremia score, median (IQR) | 1.0 (0.0-3.0) | 2.0 (0.0-4.0) | 0.161 |  | 2.0 (1.0-3.0) | 2.0 (0.0-4.0) | 0.561 |
| APACHE II score, median (IQR) | 11.0 (8.0-17.0) | 13.5 (8.3-19.8) | 0.092 |  | 13.0 (9.0-18.0) | 13.5 (8.3-19.8) | 0.479 |

Data are presented as number (%) of patients, unless stated otherwise.

IQR, interquartile range; APACHE, Acute Physiology And Chronic Health Evaluation; N/A, not applicable

^a^Intra-abdominal infection was defined as infections of single organs of abdomen with or without extension into the peritoneal space with exclusion of liver abscess

^b^Immunosuppression was defined as meeting one of the following criteria: neutropenia, use of corticosteroids, or receiving chemotherapy.

^c^Endotracheal tube was defined as patient being intubated at the onset of bacteremia.

^d^Including cefazolin and cefuroxime

^e^Including cefoperazone, ceftriaxone, cefotaxime, cefepime, and cefpirome

^f^Including amoxicillin/clavulanate, ampicillin/sulbactam, piperacillin/tazobactam, and ticarcillin/clavulanate

^g^Including ertapenem, imipenem, meropenem, and doripenem

^h^Including ciprofloxacin, levofloxacin, and moxifloxacin

^i^Including amikacin, gentamicin and isepamicin.

^j^Including vancomycin and teicoplanin

**Table S7**. Comparison of clinical outcomes in patients infected with hypervirulent *K. pneumoniae* strains that exhibited wild-type antibiotic susceptibility receiving non-carbapenems versus carbapenems as definitive antibiotic

|  | Original cohort | | |  | Propensity score-matched cohort | | |
| --- | --- | --- | --- | --- | --- | --- | --- |
|  | Non-carbapenems (n=161) | Carbapenems (n=34) | *p* value |  | Non-carbapenems (n=68) | Carbapenems (n=34) | *p* value |
| Appropriate empirical antimicrobial therapy | 159 (98.8) | 34 (100.0) | 0.514 |  | 68 (100.0) | 34 (100.0) | N/A |
| Length of hospital stay, median (IQR), days | 17.0 (12.0-32.0) | 32.0 (14.5-44.0) | 0.037 |  | 19.0 (12.8-37.0) | 32.0 (14.5-44.0) | 0.130 |
| 14-day mortality | 12 (7.5) | 5 (14.7) | 0.173 |  | 6 (8.8) | 5 (14.7) | 0.573 |
| 28-day mortality | 17 (10.6) | 8 (23.5) | 0.040 |  | 7 (10.3) | 8 (23.5) | 0.138 |
| In-hospital mortality | 23 (14.3) | 11 (32.4) | 0.012 |  | 10 (14.7) | 11 (32.4) | 0.069 |

Data are presented as number (%) of patients, unless stated otherwise.

IQR, interquartile range

**Table S8.** Multivariate logistic regression analysis of predictors for 28-day mortality in propensity score-matched patients with hypervirulent *K. pneumoniae* bacteremia

| Variables | Univariate analysis  OR (95% CI) | *p* value | Multivariate analysis  OR (95% CI) | *p* value |
| --- | --- | --- | --- | --- |
| Definitive antibiotic |  |  |  |  |
| Non-carbapenems | 1.00 (reference) |  | 1.00 (reference) |  |
| Carbapenems | 1.92 (0.81-4.55) | 0.133 | 1.25 (0.45-3.38) | 0.654 |
| Malignancy | 2.49 (1.06-6.00) | 0.037 | 1.90 (0.71-5.14) | 0.199 |
| Immunosuppression | 3.60 (1.44-8.89) | 0.005 | 2.36 (0.84-6.53) | 0.098 |
| Prior antibiotic exposure | 4.25 (1.78-10.75) | 0.001 | 2.40 (0.86-6.81) | 0.095 |
| Source control | 0.32 (0.09-0.90) | 0.047 | 0.64 (0.17-2.06) | 0.478 |
| APACHE II score | 1.07 (1.02-1.12) | 0.008 | 1.06 (1.00-1.12) | 0.055 |

APACHE, Acute Physiology And Chronic Health Evaluation

**Table S9**. Multivariate logistic regression analysis of predictors for 28-day mortality in propensity score-matched patients with hypervirulent *K. pneumoniae* bacteremia with an APACHE II score of 15 or higher

| Variables | Univariate analysis  OR (95% CI) | *p* value | Multivariate analysis  OR (95% CI) | *p* value |
| --- | --- | --- | --- | --- |
| Definitive antibiotic |  |  |  |  |
| Non-carbapenems | 1.00 (reference) |  | 1.00 (reference) |  |
| Carbapenems | 1.95 (0.68-5.55) | 0.206 | 2.01 (0.46-8.90) | 0.347 |
| Male | 0.22 (0.07-0.62) | 0.005 | 0.08 (0.02-0.32) | <0.001 |
| Community-acquired bacteremia | 0.20 (0.03-0.77) | 0.040 | 0.18 (0.02-1.55) | 0.131 |
| Prior antibiotic exposure | 4.14 (1.44-13.14) | 0.010 | 1.88 (0.37-11.00) | 0.453 |
| Chronic obstructive lung disease | 7.38 (1.32-56.77) | 0.028 | 12.50 (1.49-153.66) | 0.028 |
| Immunosuppression | 3.40 (1.09-10.58) | 0.033 | 1.25 (0.25-5.57) | 0.776 |

**Table S10.** Multivariate logistic regression analysis of predictors for 28-day mortality in propensity score-matched patients infected with hypervirulent *K. pneumoniae* strains that exhibited wild-type antibiotic susceptibility

| Variables | Univariate analysis  OR (95% CI) | *p* value | Multivariate analysis  OR (95% CI) | *p* value |
| --- | --- | --- | --- | --- |
| Definitive antibiotic |  |  |  |  |
| Non-carbapenems | 1.00 (reference) |  | 1.00 (reference) |  |
| Carbapenems | 2.68 (0.88-8.40) | 0.082 | 2.13 (0.54-8.57) | 0.272 |
| Immunosuppression | 5.49 (1.73-18.01) | 0.003 | 5.19 (1.27-23.07) | 0.023 |
| Nasogastric/Nasojejunal tube at onset of bacteremia | 3.48 (1.03-11.30) | 0.039 | 1.13 (0.21-5.15) | 0.871 |
| Prior antibiotic exposure | 6.66 (2.11-22.52) | 0.001 | 2.85 (0.66-11.90) | 0.150 |
| APACHE II score | 1.10 (1.02-1.18) | 0.013 | 1.09 (1.00-1.19) | 0.037 |

APACHE, Acute Physiology And Chronic Health Evaluation
